# Coffee consumption by type and risk of digestive cancer: a large prospective cohort study

**DOI:** 10.1038/s41416-019-0465-y

**Published:** 2019-05-01

**Authors:** Kim Tu Tran, Helen G. Coleman, Úna C. McMenamin, Chris R. Cardwell

**Affiliations:** 1 Cancer Epidemiology Research Group, Centre for Public Health, Queen’s University Belfast, Belfast, Northern Ireland UK; 2 Centre for Cancer Research and Cell Biology, Queen’s University Belfast, Belfast, Northern Ireland UK

**Keywords:** Gastrointestinal cancer, Epidemiology, Cancer epidemiology, Cancer prevention, Hepatocellular carcinoma

## Abstract

**Background:**

Inverse associations have been observed between coffee consumption and liver cancer, but associations for other digestive cancers are unclear. Few previous studies have investigated coffee type (specifically instant or ground coffee) or a range of digestive cancer types within one cohort. We therefore investigated coffee consumption by type and digestive cancer risks in a population-based cohort.

**Methods:**

The UK Biobank captured self-reported coffee consumption and cancer-registry recorded incident digestive cancers. Hazard ratios (HRs) and 95% CIs were calculated using Cox regression. The risk of every type of digestive cancer was investigated in association with coffee consumption by dose–response and by coffee type (decaffeinated, instant and ground).

**Results:**

Over 7.5 years of follow-up, 3567 developed digestive cancer among 471,779 participants. There were 88 cases of hepatocellular carcinoma and a marked association was observed for hepatocellular carcinoma in coffee drinkers (HR 0.50, 95% CI 0.29, 0.87), which was similar for instant (HR 0.51, 95% CI 0.28, 0.93) and ground coffee (HR 0.47, 95% CI 0.20, 1.08). We did not observe significant consistently reduced risks of other individual digestive cancers amongst coffee drinkers.

**Conclusions:**

We found some evidence that coffee consumption was inversely associated with hepatocellular carcinoma which was similar by coffee type.

## Background

Coffee is one of the most commonly consumed beverages worldwide.^1^ Coffee is mostly consumed as instant or ground with the type of coffee consumed varying between countries.^2^ Instant and ground coffee have different chemical compounds, reflecting the processes involved in their production. For instance, instant coffee is reported to have higher concentrations of caffeine, polyphenols and chlorogenic acid, which contribute to antioxidant activity.^3,4^ Instant coffee receipt has also been shown to reduce the size and number of neoplastic lesions, compared with conventional coffee or caffeine only, in an experimental study in rats.^5^ However, instant coffee also contains twice as much acrylamide as ground coffee,^6^ a substance which was classified in group 2A as a probable human carcinogen,^7^ and about which there has been recent controversy regarding potential cancer risk in humans.^8,9^

Various meta-analyses have been conducted into the association between coffee consumption and risk of digestive cancers. A recent meta-analysis by the World Cancer Research Fund showed no association between coffee consumption and colorectal cancer risk,^10^ but another recent review observed small reduced risks of colorectal cancer for high daily intakes of coffee (≥5 cups per day).^11^ Other meta-analyses have not found associations between coffee consumption and gastric^12^ or oesophageal cancer risk.^13^ In contrast, there is evidence of an inverse association between coffee consumption and liver cancer, with two meta-analyses demonstrating that every one cup per day increase is associated with a 14–15% reduced risk of liver cancer.^14,15^

Despite this accumulating research, few previous studies have investigated a range of digestive cancers within the same cohort. To the best of our knowledge, most studies have investigated consumption of any coffee type, caffeinated coffee or decaffeinated coffee,^15–17^ and only one study investigated instant coffee,^18^ despite its biochemical compound differences, with respect to digestive cancer risk. Therefore, we aimed to investigate the association between coffee consumption and the risk of digestive cancers by type of coffee (decaffeinated, instant or ground) within a large prospective UK cohort.

## Methods

### Data source

The UK Biobank contains ~500,000 volunteer participants aged 40–69 from England, Scotland and Wales recruited from 2006 to 2010.^19^ A wide range of data were collected, including lifestyle, environment, medical history and physical measures, along with biological samples. The UK Biobank is linked to cancer-registry data from the Health and Social Care Information Centre (in England and Wales) and the National Health Service Central Register (in Scotland). Cancer registries in the United Kingdom have high completeness.^20^ The UK Biobank has ethical approval from the North West Multi-Centre Research Ethics Committee. All participants provided written informed consent.

### Study design

A prospective cohort study was conducted among participants in the UK Biobank. Patients diagnosed with cancers of the digestive tract were identified using cancer-registry records (based upon ICD 10 codes: oesophagus C15, including oesophageal squamous cell carcinoma and oesophageal adenocarcinoma; based upon International Classification of Diseases for Oncology codes: stomach C16; small intestine C17; colon C18; rectal and anal cancer, including rectosigmoid junction cancer C19, rectum cancer C20 and anal cancer C21; liver C22, hepatocellular carcinoma C22.0 and intrahepatic bile duct C22.1; gallbladder and extrahepatic bile duct C23–C24; pancreas C25) up to September 30, 2014. Participants with any cancer diagnosis prior to baseline or in the year after baseline were excluded (to avoid reverse causation). Consequently, cohort participants were followed from 1 year after baseline, until the date of cancer diagnosis or censoring (on the earliest of the date of death, date of other cancer diagnosis or September 30, 2014).

### Exposure assessment

Coffee consumption was assessed at baseline. Participants were asked their average intake of coffee in the last year: “how many cups of coffee do you drink each day”, and the most common type of coffee used (decaffeinated, instant, ground or other type).

### Covariates

Covariates were determined from patient interview or touchscreen at baseline. These included age, sex, education (the highest qualification achieved), Townsend deprivation scores (a socio-economic measure based on area of residence)^21^ and comorbidities (high cholesterol, diabetes, hypertension, angina, myocardial infarction, stroke, peptic ulcer disease, hepatitis, cirrhosis and gallstones). Lifestyle factors, including physical activity (metabolic equivalents (METs) score was calculated based on the number of days per week with more than 10 min of walking, moderate or vigorous physical activity),^22^ fruit and vegetable intake (portions per day), tea intake (number of cups per day), smoking status (never smoker, previous smoker or current smoker) and alcohol consumption (never, <1 day per week, 1–2 days per week, 3–4 days per week or >4 days per week), were also ascertained. Body mass index (BMI) in kg/m^2^ (categorized as under- or normal weight [<25], overweight [25 to <30] or obese [30 or higher]) was calculated from height and weight measurements recorded at baseline by trained research staff. Categorical characteristics were compared by coffee consumption (yes/no) using chi-square tests.

### Statistical analysis

The UK Biobank cohort was analysed using Cox regression with age as the underlying timescale (individuals were considered at risk from birth and under observation from age at baseline, left truncated) to calculate hazard ratios (HR) and 95% confidence intervals (CI) for coffee use and risk of all digestive cancers, as well as by cancer type. Dose–response analyses were conducted by increasing cups of coffee intake. In adjusted analyses, the model contained age at baseline, sex, deprivation, education, BMI, alcohol, smoking, fruit and vegetable intake, tea intake, physical activity and comorbidities at baseline (including high cholesterol, hypertension, diabetes, angina, myocardial infarction, stroke, peptic ulcer disease, hepatitis, cirrhosis and gallstones). Estimates of coffee intake by the number of cups per day and by type in association with digestive cancer were calculated. Likelihood ratio test was used to test for heterogeneity in the risk of cancer by coffee type, and in this comparison, non-users of coffee were excluded. Tests for differences in the association between coffee consumption and cancer by sex, BMI categories, alcohol consumption and smoking status, were conducted for hepatocellular carcinoma (HCC) by including interaction terms within Cox regression models.

Sensitivity analyses were performed for every site of digestive cancer and all digestive cancers by repeating the analyses starting the follow-up at 2 years after baseline (to remove cancers within 2 years, which could have influenced coffee consumption at baseline). A separate analysis was conducted for the association between coffee intake and risk of digestive cancer, in addition adjusting for a more detailed smoking status (never smoker, previous smoker [years of cessation more than 20, 10–20 and less than 10 years] or current smoker [less than 10 cigarettes, 10–20 cigarettes and more than 20 cigarettes per day]).

Figures were drawn to visualise the dose–response pattern of coffee and coffee by type in association with digestive cancer.

## Results

A total of 471,779 participants were included in the study, following the exclusion of 30,839 participants with a previous history of cancer prior to baseline or in the year after baseline. Within the cohort, 365,157 participants (77.4%) reported drinking coffee, 104,465 participants (22.1%) reported that they did not drink coffee and in 2157 participants (0.5%) coffee consumption could not be determined. Among coffee drinkers, the type of coffee most frequently consumed was instant coffee (42.8%), followed by ground coffee (17.6%), decaffeinated coffee (14.7%) and other types reported by 1.4%.

Table 1 shows the baseline characteristics of participants by coffee consumption. People who drank coffee, compared with those who did not, were more likely to be older, male, from less-deprived areas and have higher education levels. They were also more likely to be previous or current smokers, consume higher levels of alcohol, have high cholesterol and were less likely to have diabetes, cirrhosis, gallstones, and peptic ulcers compared with non-coffee drinkers.

**Table 1:** Baseline characteristics by coffee consumption within the UK Biobank

Characteristics	Coffee use		*p*-value
	No	Yes	
*n*	104,465 (22.1%)	365,157 (77.4%)	
*Age at baseline (years)*			
0–49	29,969 (28.7%)	84,182 (23.1%)	
50–59	36,583 (35.0%)	121,413 (33.2%)	
60–69	37,513 (35.9%)	157,846 (43.2%)	<0.001
70+	400 (0.4%)	1716 (0.5%)	
Male	44,004 (42.1%)	172,206 (47.2%)	<0.001
*Deprivation*			
1 (least deprived)	17,825 (17.1%)	76,482 (20.9%)	
2	18,848 (18.1%)	75,006 (20.5%)	
3	19,880 (19.0%)	73,840 (20.2%)	<0.001
4	21,945 (21.0%)	72,053 (19.7%)	
5 (most deprived)	25,828 (24.7%)	67,325 (18.4%)	
Missing	139 (0.1%)	451 (0.1%)	
*Education*			
College or university degree	27,872 (26.7%)	124,155 (34.0%)	
A level/AS level or equivalent	10,880 (10.4%)	41,252 (11.3%)	
O level/GCSEs or equivalent	22,221 (21.3%)	76,381 (20.9%)	
CSEs or equivalent	6963 (6.7%)	18,690 (5.1%)	<0.001
NVQ or HND or HNC equivalent	7348 (7.0%)	23,358 (6.4%)	
Other professional qualifications	5113 (4.9%)	18,800 (5.1%)	
None of the above	21,679 (20.7%)	56,480 (15.5%)	
Missing	2389 (2.3%)	6041 (1.7%)	
*BMI*			
Normal/underweight	34,646 (33.2%)	119,867 (32.8%)	
Overweight	42,406 (40.6%)	156,214 (42.8%)	<0.001
Obese	26,636 (25.5%)	87,349 (23.9%)	
Missing	777 (0.7%)	1727 (0.5%)	
*Smoking status*			
Never	61,572 (58.9%)	195,888 (53.6%)	
Previous	32,229 (30.8%)	128,414 (35.2%)	<0.001
Current	10,235 (9.8%)	39,574 (10.8%)	
Missing	429 (0.4%)	1281 (0.4%)	
*Alcohol consumption*			
Never	15,214 (14.6%)	22,324 (6.1%)	
<1 day per week	29,697 (28.4%)	76,527 (20.9%)	
1–2 days per week	25,970 (24.9%)	95,315 (26.1%)	<0.001
3–4 days per week	18,424 (17.6%)	90,390 (24.8%)	
>4 days per week	15,045 (14.4%)	80,321 (22.0%)	
Missing	115 (0.1%)	280 (0.1%)	
*Fruit and vegetable intake*			
<2.5 portions per day	22,939 (21.9%)	68,417 (18.7%)	
2.5–5 portions per day	39,898 (38.2%)	149,040 (40.8%)	<0.001
>=5 portions per day	38,251 (36.6%)	138,186 (37.8%)	
Missing	3377 (3.3%)	9514 (2.6%)	
*Physical activity*			
Low	14,539 (13.9%)	47,718 (13.1%)	
Moderate	31,313 (30.0%)	119,318 (32.7%)	
High	32,617 (31.2%)	116,169 (31.8%)	<0.001
Missing	25,996 (24.9%)	81,952 (22.4%)	
*Comorbidities*			
High cholesterol	12,172 (11.7%)	44,859 (12.3%)	<0.001
Diabetes	5763 (5.5%)	17,856 (4.9%)	<0.001
Hepatitis	520 (0.5%)	1856 (0.5%)	0.45
Cirrhosis	129 (0.12%)	348 (0.10%)	0.041
Gallstones	1775 (1.7%)	5700 (1.5%)	0.002
Peptic ulcers	1599 (1.5%)	4090 (1.1%)	<0.001

*BMI* body mass index, *CSE* Certificate of Secondary Education, *GCSE* General Certificate of Secondary Education, *HNC* Higher National Certificate, *HND* Higher National Diploma, *NVQ* National Vocational Qualification

Over 7.5 years of follow-up, 3567 digestive cancer cases were detected. The associations between any type of coffee consumption and risk of specific digestive cancers are presented in Table 2. In adjusted analysis, any coffee consumption was only associated with HCC (adjusted HR: 0.50, 95% CI: 0.29–0.87) and was not significantly associated with other cancers. The results were largely similar in dose–response analyses of increasing the number of coffee cups consumed per day compared with non-coffee drinkers. The association of oesophageal squamous cell carcinoma and coffee intake followed a dose– response relationship (adjusted HR per cup increase 1.08, 95% CI: 1.02–1.16) and an increased risk was observed in those drinking more than five cups (adjusted HR: 2.88, 95% CI: 1.20–6.92). There was some evidence of reductions in HCC risk with increasing the categories of coffee cups consumed (e.g., adjusted HR for ≥5 cups: 0.44, 95% CI: 0.17–1.12 and p for trend = 0.07), as well as per cup increase (adjusted HR: 0.87, 95% CI: 0.76–1.01).

**Table 2:** The association between coffee intake by the number of cups per day and digestive cancer within the UK Biobank

			Coffee intake (cups/day)			HR per cup increase	p-trend
	0	Any	>0–2	3–4	≥5		
*n*	104,465	365,157	215,739	97,022	52,396		
*Oesophageal cancer*							
Cases	74	279	146	72	61		
Unadjusted HR	1.00	0.97 (0.75, 1.25)	0.84 (0.63, 1.11)	0.93 (0.67, 1.29)	1.60 (1.14, 2.24)	1.07 (1.03, 1.10)	<0.001
Adjusted HR	1.00	1.15 (0.83, 1.61)	1.11 (0.77, 1.58)	1.08 (0.71, 1.65)	1.47 (0.94, 2.30)	1.03 (0.98, 1.07)	0.28
*Oesophageal squamous cell carcinoma*							
Cases	15	61	28	15	18		
Unadjusted HR	1.00	1.05 (0.60, 1.86)	0.80 (0.42, 1.51)	0.97 (0.47, 1.99)	2.34 (1.18, 4.64)	1.09 (1.03, 1.15)	0.001
Adjusted HR	1.00	1.26 (0.59, 2.67)	0.94 (0.41, 2.14)	1.10 (0.42, 2.87)	2.88 (1.20, 6.92)	1.08 (1.02, 1.16)	0.01
*Oesophageal adenocarcinoma*							
Cases	55	204	110	52	42		
Unadjusted HR	1.00	0.94 (0.70, 1.27)	0.84 (0.61, 1.17)	0.90 (0.61, 1.32)	1.47 (0.98, 2.20)	1.06 (1.01, 1.11)	0.008
Adjusted HR	1.00	1.21 (0.81, 1.80)	1.25 (0.83, 1.89)	1.07 (0.65, 1.76)	1.25 (0.72, 2.15)	1.00 (0.93, 1.07)	0.96
*Gastric cancer*							
Cases	65	184	99	55	30		
Unadjusted HR	1.00	0.72 (0.55, 0.96)	0.65 (0.47, 0.88)	0.81 (0.56, 1.16)	0.90 (0.58, 1.39)	1.00 (0.95, 1.07)	0.79
Adjusted HR	1.00	0.98 (0.68, 1.41)	0.88 (0.59, 1.30)	1.14 (0.73, 1.79)	1.18 (0.70, 1.98)	1.03 (0.97, 1.09)	0.30
*Small intestinal cancer*							
Cases	14	63	31	20	12		
Unadjusted HR	1.00	1.18 (0.66, 2.11)	0.97 (0.51, 1.82)	1.40 (0.71, 2.78)	1.68 (0.78, 3.64)	1.05 (0.96, 1.14)	0.27
Adjusted HR	1.00	1.39 (0.60, 2.87)	1.12 (0.49, 2.58)	1.67 (0.67, 4.18)	1.65 (0.56, 4.78)	1.02 (0.90, 1.17)	0.65
*Colon cancer*							
Cases	320	1141	666	340	135		
Unadjusted HR	1.00	0.93 (0.82, 1.05)	0.90 (0.79, 1.03)	1.04 (0.89, 1.21)	0.82 (0.67, 1.00)	0.98 (0.95, 1.00)	0.16
Adjusted HR	1.00	0.91 (0.78, 1.05)	0.89 (0.76, 1.05)	0.97 (0.80, 1.18)	0.87 (0.68, 1.11)	0.97 (0.93, 1.01)	0.15
*Rectal and anal cancer*							
Cases	176	610	361	155	94		
Unadjusted HR	1.00	0.91 (0.77, 1.08)	0.90 (0.76, 1.08)	0.87 (0.70, 1.08)	1.04 (0.81, 1.33)	1.01 (0.98, 1.04)	0.37
Adjusted HR	1.00	0.88 (0.72, 1.08)	0.90 (0.72, 1.11)	0.81 (0.62, 1.06)	0.95 (0.70, 1.30)	1.00 (0.96, 1.04)	0.90
*Liver cancer*							
Cases	48	134	82	32	20		
Unadjusted HR	1.00	0.72 (0.52, 1.00)	0.74 (0.52, 1.05)	0.65 (0.41, 1.01)	0.81 (0.48, 1.37)	0.96 (0.89, 1.04)	0.34
Adjusted HR	1.00	0.87 (0.58, 1.30)	0.88 (0.57, 1.36)	0.87 (0.51, 1.48)	0.83 (0.45, 1.55)	0.97 (0.89, 1.05)	0.44
*Hepatocellular carcinoma*							
Cases	26	62	39	14	9		
Unadjusted HR	1.00	0.61 (0.39, 0.97)	0.64 (0.39, 1.05)	0.52 (0.27, 1.00)	0.68 (0.32, 1.45)	0.93 (0.82, 1.04)	0.22
Adjusted HR	1.00	0.50 (0.29, 0.87)	0.55 (0.30, 1.00)	0.44 (0.20, 0.96)	0.44(0.17, 1.12)	0.87 (0.76, 1.01)	0.07
*Intrahepatic bile duct carcinoma*							
Cases	16	56	36	14	6		
Unadjusted HR	1.00	0.90 (0.52, 1.57)	0.97 (0.53, 1.74)	0.84 (0.41, 1.73)	0.72 (0.28, 1.85)	0.95 (0.84, 1.07)	0.43
Adjusted HR	1.00	1.49 (0.71, 3.11)	1.47 (0.68, 3.18)	1.80 (0.74, 4.34)	1.06 (0.32, 3.48)	1.00 (0.88, 1.14)	0.90
*Gallbladder and extrahepatic bile duct carcinoma*							
Cases	14	81	47	20	14		
Unadjusted HR	1.00	1.48 (0.84, 2.61)	1.43 (0.78, 2.59)	1.37 (0.69, 2.71)	1.93 (0.92, 4.06)	1.05 (0.96, 1.13)	0.23
Adjusted HR	1.00	1.63 (0.81, 3.27)	1.52 (0.74, 3.15)	1.56 (0.67, 3.64)	2.40 (0.98, 5.90)	1.06 (0.97, 1.15)	0.15
*Pancreatic cancer*							
Cases	81	303	171	83	49		
Unadjusted HR	1.00	0.95 (0.75, 1.22)	0.89 (0.68, 1.16)	0.98 (0.72, 1.33)	1.17 (0.82, 1.67)	1.03 (0.98, 1.07)	0.17
Adjusted HR	1.00	0.89 (0.66, 1.20)	0.86 (0.63, 1.18)	0.88 (0.60, 1.28)	1.08 (0.71, 1.65)	1.02 (0.97, 1.06)	0.44
*All digestive cancers*							
Cases	784	2767	1,581	773	413		
Unadjusted HR	1.00	0.91 (0.84, 0.99)	0.87 (0.80, 0.95)	0.96 (0.87, 1.06)	1.02 (0.91, 1.15)	1.01 (0.99, 1.02)	0.17
Adjusted HR	1.00	0.95 (0.86, 1.05)	0.92 (0.83, 1.02)	0.98 (0.87, 1.11)	1.05 (0.90, 1.21)	1.00 (0.98, 1.02)	0.53

The model contains age at baseline, sex, deprivation, education, BMI, alcohol, smoking, fruit and vegetable intake, tea intake, physical activity and comorbidities at baseline (including high cholesterol, hypertension, diabetes, angina, myocardial infarction, stroke, peptic ulcer disease, hepatitis, cirrhosis and gallstones)

Associations between coffee consumption by type and risk of specific digestive cancers are presented in Table 3. The reduction of HCC risk ranged from 41 to 53% for different types of coffee, which was most apparent for instant coffee (adjusted HR: 0.51, 95% CI: 0.28–0.93); however, there was no evidence of the difference in the risk of HCC by type of coffee (*p* = 0.53). The risk of pancreatic cancer was reduced by 34% in users of decaffeinated coffee compared with non-coffee drinkers, but this did not reach statistical significance (adjusted HR: 0.66, 95% CI: 0.43–1.01). No significant associations were observed for any coffee type and risk of stomach, oesophagus, small intestine, colon, rectal and anal cancer or intrahepatic bile duct carcinoma (Table 3). The risk of gallbladder and extrahepatic bile duct carcinoma was consistently increased with consumption of any type of coffee, but the only significant increase in risk was observed for decaffeinated coffee (HR 2.44, 95% CI: 1.10–5.38).

**Table 3:** The association between coffee type and digestive cancer within the UK Biobank

		Most common type of coffee used				
	Non-use	Decaffeinated	Instant	Ground	Other types	Heterogeneity p^a^
*n*	104,465	69,363	201,912	82,865	6674	
*Oesophageal cancer*						
Cases	74	53	166	46	7	
Unadjusted HR	1.00	0.91 (0.64, 1.30)	1.03 (0.78, 1.36)	0.76 (0.52, 1.09)	1.43 (0.66, 3.11)	0.18
Adjusted HR	1.00	1.27 (0.82, 1.98)	1.12 (0.78, 1.59)	1.08 (0.78, 1.59)	1.58 (0.56, 4.41)	0.63
*Oesophageal squamous cell carcinoma*						
Cases	15	14	36	8	<5	
Unadjusted HR	1.00	1.21 (0.58, 2.52)	1.12 (0.61, 2.05)	0.65 (0.27, 1.54)	2.04 (0.46, 8.92)	0.54
Adjusted HR	1.00	1.27 (0.46, 3.51)	1.16 (0.52, 2.58)	1.03 (0.38, 2.83)	4.17 (0.89,19.46)	0.87
*Oesophageal adenocarcinoma*						
Cases	55	36	122	35	6	
Unadjusted HR	1.00	0.83 (0.54, 1.26)	1.01 (0.73, 1.39)	0.77 (0.50, 1.18)	1.64 (0.71, 3.83)	0.60
Adjusted HR	1.00	1.33 (0.79, 2.24)	1.18 (0.78, 1.80)	1.17 (0.69, 1.97)	1.65 (0.50, 5.41)	0.63
*Gastric cancer*						
Cases	65	29	117	31	<5	
Unadjusted HR	1.00	0.57 (0.37, 0.88)	0.83 (0.61, 1.12)	0.59 (0.38, 0.90)	0.93 (0.34, 2.56)	0.13
Adjusted HR	1.00	0.88 (0.52, 1.48)	1.04 (0.70, 1.52)	0.87 (0.52, 1.45)	0.90 (0.22, 3.76)	0.69
*Small intestinal cancer*						
Cases	14	11	35	12	<5	
Unadjusted HR	1.00	1.04 (0.47, 2.30)	1.18 (0.63, 2.20)	1.06 (0.49, 2.29)	2.20 (0.50, 9.70)	0.82
Adjusted HR	1.00	1.70 (0.65, 4.41)	1.07 (0.46, 2.51)	1.16 (0.42, 3.15)	4.39 (0.93, 20.8)	0.34
*Colon cancer*						
Cases	320	220	650	223	22	
Unadjusted HR	1.00	0.90 (0.76, 1.07)	0.95 (0.83, 1.09)	0.86 (0.72, 1.02)	1.05 (0.68, 1.62)	0.51
Adjusted HR	1.00	0.95 (0.78, 1.17)	0.91 (0.77, 1.07)	0.84 (0.68, 1.04)	1.21 (0.73, 2.02)	0.50
*Rectal and anal cancer*						
Cases	176	103	371	119	8	
Unadjusted HR	1.00	0.77 (0.61, 0.99)	0.99 (0.83, 1.19)	0.83 (0.66, 1.05)	0.69 (0.34, 1.42)	0.06
Adjusted HR	1.00	0.83 (0.62, 1.11)	0.95 (0.76, 1.18)	0.80 (0.60, 1.05)	0.56 (0.20, 1.53)	0.39
*Liver cancer*						
Cases	48	23	80	26	<5	
Unadjusted HR	1.00	0.62 (0.37, 1.02)	0.77 (0.54, 1.11)	0.67 (0.41, 1.07)	0.95 (0.29, 3.06)	0.72
Adjusted HR	1.00	0.82 (0.45, 1.49)	0.91 (0.59, 1.41)	0.84 (0.47, 1.50)	0.58 (0.07, 4.24)	0.92
*Hepatocellular carcinoma*						
Cases	26	11	37	11	<5	
Unadjusted HR	1.00	0.54 (0.27, 1.10)	0.66 (0.40, 1.09)	0.52 (0.25, 1.05)	1.17 (0.28, 4.95)	0.71
Adjusted HR	1.00	0.59 (0.25, 1.37)	0.51 (0.28, 0.93)	0.47 (0.20, 1.08)	Too small	0.53
*Intrahepatic bile duct carcinoma*						
Cases	16	9	33	12	<5	
Unadjusted HR	1.00	0.72 (0.32, 1.64)	0.95 (0.52, 1.73)	0.92 (0.43, 1.94)	0.95 (0.12, 7.17)	0.91
Adjusted HR	1.00	0.97 (0.38, 2.43)	1.27 (0.63, 2.55)	1.46 (0.63, 3.35)	1.48 (0.19,11.54)	0.91
*Gallbladder and extrahepatic bile duct carcinoma*						
Cases	14	25	34	18	<5	
Unadjusted HR	1.00	2.28 (1.18, 4.39)	1.11 (0.60, 2.08)	1.57 (0.78, 3.16)	2.17 (0.78, 3.16)	0.06
Adjusted HR	1.00	2.44 (1.10, 5.38)	1.33 (0.63, 2.82)	1.84 (0.77, 4.44)	Too small	0.11
*Pancreatic cancer*						
Cases	81	46	178	69	<5	
Unadjusted HR	1.00	0.72 (0.50, 1.03)	1.00 (0.77, 1.31)	1.04 (0.75, 1.43)	0.93 (0.37, 2.30)	0.17
Adjusted HR	1.00	0.66 (0.43, 1.03)	0.95 (0.69, 1.29)	0.99 (0.68, 1.45)	0.81 (0.25, 2.60)	0.31
*All digestive cancers*						
Cases	784	507	1614	536	53	
Unadjusted HR	1.00	0.84 (0.75, 0.94)	0.96 (0.88, 1.04)	0.84 (0.75, 0.93)	1.03 (0.78, 1.36)	0.007
Adjusted HR	1.00	0.95 (0.83, 1.09)	0.97 (0.87, 1.07)	0.89 (0.78, 1.02)	1.03 (0.72, 1.47)	0.61

The model contains age at baseline, sex, deprivation, education, BMI, alcohol, smoking, fruit and vegetable intake, tea intake, physical activity and comorbidities at baseline (including high cholesterol, hypertension, diabetes, angina, myocardial infarction, stroke, peptic ulcer disease, hepatitis, cirrhosis and gallstones)

^a^P-value for likelihood ratio test comparing cancer risk by coffee type (decaffeinated, instant, ground and other types)

After excluding the 2 years following baseline, the association between increasing the number of coffee cups consumed per day and digestive cancer risk showed similar results to the main analysis (Supplementary Table 1). In similar analysis by coffee type, associations were little altered for any digestive cancer site, except for gallbladder and extrahepatic bile duct carcinoma, which attenuated and became non-significant (Supplementary Table 2).

An additional analysis was conducted for the association between coffee intake and risk of digestive cancer, in addition adjusting for a more detailed smoking status. The association was observed to be similar to the main analysis (Supplementary Table 3). Analysis for HCC stratifying by sex, BMI, alcohol consumption and smoking status found similar association to the main analysis (Supplementary Table 4). No interaction was found.

Supplementary Figures 1 and 2 were drawn to visualise the dose–response pattern of coffee and coffee by type in association with digestive cancer.

## Discussion

In a large prospective study, we found that a reduced risk of HCC with coffee consumption, which was consistent with dose–response analysis and by coffee type, was apparent for instant coffee.

Our study is consistent with the inverse association between coffee consumption and HCC risk that has been judged as “convincing” evidence in the 2018 World Cancer Research Fund report,^15^ confirming previous individual studies^16,17,23^ and meta-analyses.^24,25^ Whilst we did not show marked differences in the inverse association between HCC and coffee consumption by type of coffee, an inverse association was apparent for instant coffee. Some previous studies have investigated caffeinated and decaffeinated coffee,^16,17,23^ but no previous studies, to date, have explored the association of instant coffee consumption and HCC.

The underlying biological mechanisms for a reduced risk of HCC with coffee consumption, especially with instant coffee, are not well known, but some mechanisms have been proposed. Coffee has high levels of antioxidants,^26^ including phenolic acids, diterpenes like cafestol and kahweol and tocopherols.^27^ Compounds, such as phenolic acids^28,29^ and caffeine,^30^ have also been shown to have chemopreventive properties, including in liver carcinogenesis.^31^ These compounds have been shown to inhibit the proliferation of HCC cell lines in vitro and suppress the progression of HCC in vivo,^32^ while chlorogenic acid has been shown to prevent oxidative damage in hepatocytes.^33^ Instant coffee, in comparison with ground and decaffeinated coffee, has been demonstrated to have higher levels of those biochemical compounds,^3,4^ which therefore could partly explain the more marked reduced risk of HCC in our study. The underlying potential anticancer mechanism of instant coffee has also been observed in previous experimental studies. An animal study^5^ showed that mice receiving instant coffee had a reduction in the size and the number of hepatocellular neoplastic lesions, compared with mice receiving ground coffee. Instant coffee administration in rats also resulted in a significantly higher expression of bax protein,^5^ which monitors cell apoptosis and is a known tumour suppressor.^34,35^ Another study in rats demonstrated that instant coffee powder inhibited hepatoma cell proliferation, reduced metastasis and positively altered lipoprotein profiles.^36^ Therefore, it is plausible that instant coffee may have stronger anticancer effects in comparison with other coffee types.

For other individual types of digestive cancer, we did not find any consistent association with coffee consumption. Our findings of no association between coffee and colorectal cancer confirm previous findings,^10,37^ although they contrast with suggestions that high levels of coffee drinking reduced the risk of colon cancer.^11^ For small intestine and oesophageal cancer, our findings are similar to previous meta-analyses of no association with coffee consumption.^13,38^ However, our study found an increased risk of oesophageal squamous cell carcinoma with high intake of coffee, which was not consistent with the literature. A study in a Norwegian population found no association between high coffee intake and the risk of oesophageal squamous cell carcinoma.^31^ Similarly, another study found that higher intake of isoflavones, for which coffee was the major source, was not associated with the risk of oesophageal squamous cell carcinoma.^39^

For gastric cancer, the results are inconclusive. Our findings of no association between coffee and the risk of gastric cancer are in line with a recent meta-analysis;^12^ however, contrasting two previous meta-analyses showed a decrease^40^ and an increase in the risk of gastric cancer with coffee use.^41^ In one meta-analysis, no association was observed following restriction to only cohort studies,^40^ while in the other meta-analysis, the positive association between coffee consumption and gastric cancer risk attenuated and became non-significant after adjusting for risk factors, such as smoking, alcohol intake and BMI.^41^

Our study is similar to a more recent meta-analysis of four large cohort studies of female non-smokers, which demonstrated no association between coffee intake and pancreatic cancer.^42^ Our study found no association between increasing the number of coffee cups consumed and pancreatic cancer risk, but there was suggestive evidence of an inverse association between decaffeinated coffee consumption and pancreatic cancer risk, which is in contrast to a previous US study, showing no association for decaffeinated coffee.^43^ The difference in the results might be explained by other underlying differences in population behaviours relevant for pancreatic cancer aetiology, or differences in coffee consumption patterns, but is difficult to fully understand. Further investigations are warranted to explore the association between decaffeinated coffee and pancreatic cancer.

Our findings of an increased risk of gallbladder cancer with decaffeinated coffee consumption are not consistent with a previous meta-analysis of any coffee intake and biliary tract cancer risk, although the previous study did not specifically investigate decaffeinated coffee.^14^ However, our result could be owing to chance, since there was no clear dose–response relationship, and the positive finding attenuated and became non-significant in sensitivity analysis.

Acrylamide is a chemical produced by the coffee-roasting process, particularly in the production of instant coffee,^6^ classified by IARC in group 2A as a probable human carcinogen.^7^ Acrylamide has been shown to have a carcinogenic effect in animal studies;^44^ however, in epidemiological studies of humans, the association with gastrointestinal cancer is controversial. For instance, a prospective study investigating daily acrylamide intake did not find an association with oesophageal, gastric, colorectal and pancreatic cancer.^45^ In contrast, another study from the European Prospective Investigation into Cancer and Nutrition cohort found a significantly increased risk of oesophageal cancer with acrylamide intake.^46^ Our study generally provides reassurance that coffee consumption, known to be high in acrylamide, does not appear to be associated with increased gastrointestinal cancer risk.

The main strength of our study is that within the UK Biobank, information on the type of coffee most commonly consumed was available, allowing for investigation of instant or ground or decaffeinated coffee and digestive cancer risk. Second, the UK Biobank contains over 500,000 participants who were prospectively followed for up to 7.5 years, allowing sufficient statistical power to detect even relatively weak associations. We were also able to investigate the impact of coffee consumption across a number of different digestive cancer sites, therefore minimizing potential measurement error. Finally, we were able to control for various important confounders, which are associated with coffee consumption and digestive cancer risk, such as BMI, alcohol and smoking habit, physical activity, fruit and vegetable intake, tea intake and comorbidities.

However, a number of weaknesses existed in our study. First, coffee consumption could have changed over time, so misclassification of coffee consumption is possible. However, other cohorts have shown coffee consumption to remain relatively stable over time in adult populations.^47^ Second, there were small numbers of cases of certain cancers in our study (e.g., small intestine cancer, intrahepatic bile duct carcinoma, gallbladder and extrahepatic bile duct carcinoma), and therefore for these cancers, we would have limited power to detect associations with coffee consumption. Third, we did not have information on H. pylori infection; therefore, we were unable to adjust for this potential confounder in analyses of oesophageal or gastric cancer. Fourth, we did not investigate the impact of milk, non-dairy creamer and sweeteners on the association between coffee and digestive cancer risk. Milk and non-dairy creamer have been found to alter the biochemical activities of coffee by interacting with coffee components like polyphenol;^48^ however, the extent of its impact on the association of coffee and cancer risk has not yet been substantiated. Measurement error of coffee intake might have affected our ability to identify true associations with cancers. In particular, it is possible that measurement error, which is likely to dilute any real associations, both reduced the magnitude of the association between coffee intake and HCC and dragged weaker associations between coffee intake and other GI cancers to the null. Finally, as a large number of tests were conducted, increasing the likelihood of Type 1 error, significant results should be interpreted cautiously. Further studies should investigate the underlying mechanisms of the inverse association between coffee and HCC. Additional large epidemiological studies are required to confirm the role of instant coffee among other types, as well as the impact of milk, non-dairy creamer and sweeteners in the association with digestive cancers.

In conclusion, our findings suggest an inverse association between coffee consumption and hepatocellular carcinoma, which was similar by coffee types. However, whether the observed association reflects a causal relationship, and if so, the underlying mechanisms are responsible, is worthy of further investigation.

## Supplementary information

Supplementary files

## Data Availability

The UK Biobank resource is available to all bona fide researchers for all types of health-related research, which is in the public interest.

## References

[1] Statista. Global beverage sales share from 2011 to 2016, by beverage type. https://www.statista.com/statistics/232773/forecast-for-global-beverage-sales-by-beverage-type/ (accessed 8 October 2018) (2018).

[2] Euromonitor Research. Ranked: Top 25 Coffee-Drinking Countries – Fresh vs Instant. Euromonitor Int. Available at https://blog.euromonitor.com/2017/09/ranked-top-25-coffee-drinking-countries-fresh-vs-instant.html (accessed October 2018).

[3] Niseteo T, Komes D, Belščak-Cvitanović A, Horžić D, Budeč M (2012). Bioactive composition and antioxidant potential of different commonly consumed coffee brews affected by their preparation technique and milk addition. Food Chem..

[4] Brezová V, Šlebodová A, Staško A (2009). Coffee as a source of antioxidants: an EPR study. Food Chem..

[5] S. Furtado K, Polletini J, C.Dias M, A. M.Rodrigues M, F. Barbisan L (2014). Prevention of rat liver fibrosis and carcinogenesis by coffee and caffeine. Food Chem. Toxicol..

[6] Mojska H, Gielecińska I (2013). Studies of acrylamide level in coffee and coffee substitutes: influence of raw material and manufacturing conditions. Rocz. Panstw. Zakl. Hig..

[7] World Health Organisation. Health implications of acrylamide in food. (2002). Geneva: World Health Organisation.

[8] Pelucchi C, Bosetti C, Galeone C, Vecchia CLa (2015). Dietary acrylamide and cancer risk: an updated meta‐analysis. Int J. Cancer.

[9] Wierzejska R (2015). Coffee consumption vs. cancer risk - a review of scientific data. Rocz. Panstw. Zakl. Hig..

[10] Vieira AR, Abar L, Chan DSM, Vingeliene S, Polemiti E, Stevens C (2017). Foods and beverages and colorectal cancer risk: a systematic review and meta-analysis of cohort studies, an update of the evidence of the WCRF-AICR Continuous Update Project. Ann. Oncol..

[11] Gan Y, Wu J, Zhang S, Li L, Cao S, Mkandawire N (2017). Association of coffee consumption with risk of colorectal cancer: a meta-analysis of prospective cohort studies. Oncotarget.

[12] Wang A, Wang S, Zhu C, Huang H, Wu L, Wan X (2016). Coffee and cancer risk: a meta-analysis of prospective observational studies. Sci. Rep..

[13] Zhang J, Zhou B, Hao C (2018). Coffee consumption and risk of esophageal cancer incidence: a meta-analysis of epidemiologic studies. Med.

[14] Godos J, Micek A, Marranzano M, Salomone F, Rio DDel, Ray S (2017). Coffee consumption and risk of biliary tract cancers and liver cancer: a dose-response meta-analysis of prospective cohort studies. Nutrients.

[15] World Cancer Research Fund/American Institute for Cancer Research. Diet, Nutrition, Physical Activity and Cancer: a Global Perspective. Continuous Update.Project Expert Report 2018. Available at https://www.wcrf.org/dietandcancer. (accessed April 2019)

[16] Setiawan VW, Wilkens LR, Lu SC, Hernandez BY, Marchand L Le, Henderson BE (2015). Association of coffee intake with reduced incidence of liver cancer and death from chronic liver disease in the US multiethnic cohort. Gastroenterology.

[17] Petrick JL, Freedman ND, Graubard BI, Sahasrabuddhe VV, Lai GY, Alavanja MC (2015). Coffee consumption and risk of hepatocellular carcinoma and intrahepatic cholangiocarcinoma by sex: the liver cancer pooling project. Cancer Epidemiol. Biomark. Prev..

[18] Schmit SL, Rennert HS, Rennert G, Gruber SB (2016). Coffee consumption and the risk of colorectal cancer. Cancer Epidemiol. Biomark. Prev..

[19] UK Biobank. About UK Biobank. http://www.ukbiobank.ac.uk/about-biobank-uk/ (accessed 2 February 2018).

[20] Møller H, Richards S, Hanchett N, Riaz SP, Lüchtenborg M, Holmberg L (2011). Completeness of case ascertainment and survival time error in English cancer registries: impact on 1-year survival estimates. Br. J. Cancer.

[21] Townsend P, Philimore P, Beattie A (1988). Health and Deprivation: Inequality and the North..

[22] IPAQ Research Committee. Guidelines for Data Processing and Analysis of the International Physical Activity Questionnaire (IPAQ) – Short and Long Forms (2005). Available at https://sites.google.com/site/theipaq/scoring-protocol (accessed April 2019).

[23] Bamia C, Lagiou P, Jena M, Trichopoulou A, Fedirko V, Aleksandrova K (2015). Coffee, tea and decaffeinated coffee in relation to hepatocellularcarcinoma in a European population: Multicentre, prospectivecohort study. Int J. Cancer.

[24] Kennedy OJ, Roderick P, Buchanan R, Fallowfield JA, Hayes PC, Parkes J (2017). Coffee, including caffeinated and decaffeinated coffee, and the risk of hepatocellular carcinoma: a systematic review and dose–response meta-analysis. BMJ Open.

[25] Bravi F, Tavani A, Bosetti C, Boffetta P, La Vecchia C (2017). Coffee and the risk of hepatocellular carcinoma and chronic liver disease: a systematic review and meta-analysis of prospective studies. Eur. J. Cancer Prev..

[26] Yashin A, Yashin Y, Wang JY, Nemzer B (2013). Antioxidant and antiradical activity of coffee. Antioxidants (Basel).

[27] Chu Y. *Coffee: Emerging Health Benefits and Disease Prevention* 1st edn, (Wiley-Blackwell, Ames, Iowa, 2012).

[28] Belkaid A, Currie JC, Desgagnés J, Annabi B (2006). The chemopreventive properties of chlorogenic acid reveal a potential new role for the microsomal glucose-6-phosphate translocase in brain tumor progression. Cancer Cell Int.

[29] Ekbatan SS, Li XQ, Ghorbani M, Azadi B, Kubow S (2018). Chlorogenic acid and its microbial metabolites exert anti-proliferative effects, S-phase cell-cycle arrest and apoptosis in human colon cancer Caco-2 cells. Int J. Mol. Sci..

[30] Fujise Y, Okano JI, Nagahara T, Abe R, Imamoto R, Murawaki Y (2012). Preventive effect of caffeine and curcumin on hepato­carcinogenesis in diethylnitrosamine-induced rats. Int J. Oncol..

[31] Tverdal A, Hjellvik V, Selmer R (2011). Coffee intake and oral–oesophageal cancer: follow-up of 389 624 Norwegian men and women 40–45 years. Br. J. Cancer.

[32] Yan Y, Liu N, Hou N, Dong L, Li J (2017). Chlorogenic acid inhibits hepatocellular carcinoma in vitro and in vivo. J. Nutr. Biochem..

[33] Merzouk AS, Hafida M, Medjdoub A, Loukidi B, Cherrak S, Merzouk SA (2017). Alterations of hepatocyte function with free radical generators and reparation or prevention with coffee polyphenols. Free Radic. Res.

[34] Brady H, Gil-Gómez G (1998). Molecules in focus Bax. The pro-apoptotic Bcl-2 family member, Bax. Int. J. Biochem. Cell Biol..

[35] Xin M., Li R., Xie M., Park D., Owonikoko T. K., Sica G. L. et al. Small-molecule Bax agonists for cancer therapy. *Nat. Commun.* **5**, 4935 (2014). 10.1038/ncomms5935 PMC4172359 25230299

[36] Miura Y, Ono K, Okauchi R, Yagasaki K (2004). Inhibitory effect of coffee on hepatoma proliferation and invasion in culture and on tumor growth, metastasis and abnormal lipoprotein profiles in hepatoma-bearing rats. J. Nutr. Sci. Vitam..

[37] Horisaki Ken, Takahashi Kunihiko, Ito Hidemi, Matsui Shigeyuki (2018). A Dose-Response Meta-analysis of Coffee Consumption and Colorectal Cancer Risk in the Japanese Population: Application of a Cubic-Spline Model. Journal of Epidemiology.

[38] Alicandro G, Tavani A, La Vecchia C (2017). Coffee and cancer risk: a summary overview. Eur. J. Cancer Prev..

[39] Petrick JL, Steck SE, Bradshaw PT, Trivers KF, Abrahamson PE, Engel LS (2015). Dietary intake of flavonoids and oesophageal and gastric cancer: incidence and survival in the United States of America (USA). Br. J. Cancer.

[40] Xie Y, Huang S, He T, Su Y (2016). Coffee consumption and risk of gastric cancer: an updated meta-analysis. Asia Pac. J. Clin. Nutr..

[41] Deng W, Yang H, Wang J, Cai J, Bai Z, Song J (2016). Coffee consumption and the risk of incident gastric cancer--a meta-analysis of prospective cohort studies. Nutr. Cancer.

[42] Zhou CD, Kuan AS, Reeves GK, Green J, Floud S, Beral V (2018). Coffee and pancreatic cancer risk among never-smokers in the UK prospective Million Women Study. Int J. Cancer.

[43] Guertin KA, Freedman ND, Loftfield E, Stolzenberg-Solomon RZ, Graubard BI, Sinha R (2015). A prospective study of coffee intake and pancreatic cancer: results from the NIH-AARP Diet and Health Study. Br. J. Cancer.

[44] Chen D, Liu H, Wang E, Yan H, Ye H, Yuan Y (2018). Toxicogenomic evaluation of liver responses induced by acrylamide and glycidamide in male mouse liver. Gen. Physiol. Biophys..

[45] Hogervorst JGF, Schouten LJ, Konings EJM, Goldbohm RA, Brandt PAvanden (2008). Dietary acrylamide intake is not associated with gastrointestinal cancer risk. J. Nutr..

[46] Lujan-Barroso L, González CA, Slimani N, Obón-Santacana M, Ferrari P, Freisling H (2014). Dietary intake of acrylamide and esophageal cancer risk in the European Prospective Investigation into Cancer and Nutrition cohort. Cancer Causes Control.

[47] Hildebrand JS, Patel AV, McCullough ML, Gaudet MM, Chen AY, Hayes RB (2012). Coffee, tea, and fatal oral/pharyngeal cancer in a large prospective US cohort. Am. J. Epidemiol..

[48] Duarte GS, Farah A (2011). Effect of simultaneous consumption of milk and coffee on chlorogenic acids’ bioavailability in humans. J. Agric Food Chem..

