# Supplementary material for: Coffee consumption by type and risk of digestive cancer: a large prospective cohort study
Source: Br J Cancer. 2019 May 1;120(11):1059–66. doi: 10.1038/s41416-019-0465-y (PMC6738036; doi:10.1038/s41416-019-0465-y)
Supplement: Supplementary file 1 — Supplementary files [file 41416_2019_465_MOESM1_ESM.docx]

| Supplementary Table 1: Sensitivity analysis by excluding two years after baseline for association between coffee intake by number of cups per day and digestive cancer within UK Biobank | | | | | | | | | | | | |
| --- | --- | --- | --- | --- | --- | --- | --- | --- | --- | --- | --- | --- |
|  | | |  | | | | Coffee intake (cups/day) | | | | HR per cup increase | p trend |
|  | 0 | | | | Any | >0 to 2 | | 3 to 4 | | ≥5 |  |  |
| *n* | 103 560 | | | | 361 910 | 213 775 | | 96 175 | | 51 960 |  |  |
|  |  | | | |  |  | |  | |  |  |  |
| Oesophageal cancer | | | | | | | | | | | | |
| Cases | 58 | | | 228 | | 118 | | 59 | | 51 |  |  |
| Adjusted HR | 1.00 | | | 1.29 (0.88, 1.89) | | 1.21 (0.80, 1.81) | | 1.24 (0.78, 1.98) | | 1.76 (1.08, 2.87) | 1.04 (0.98, 1.10) | 0.14 |
|  |  | | |  | |  | |  | |  |  |  |
| Oesophageal squamous cell carcinoma | | | | | | | |  | |  |  |  |
| Cases | 11 | | | 51 | | 24 | | 13 | | 14 |  |  |
| Adjusted HR | 1.00 | | | 1.72 (0.70, 4.20) | | 1.26 (0.48, 3.29) | | 1.68 (0.57, 4.89) | | 3.77 (1.36,10.41) | 1.09 (1.02,1.16) | 0.006 |
|  |  | | |  | |  | |  | |  |  |  |
| Oesophageal adenocarcinoma | | | | | |  | |  | |  |  |  |
| Cases | 44 | | | 166 | | 88 | | 42 | | 36 |  |  |
| Adjusted HR | 1.00 | | | 1.28 (0.82, 2.00) | | 1.29 (0.81, 2.06) | | 1.14 (0.65, 1.99) | | 1.45 (0.80, 2.62) | 1.01 (0.94, 1.08) | 0.70 |
|  |  | | |  | |  | |  | |  |  |  |
| Gastric cancer | | | | | | | | | | | | |
| Cases | 52 | | | 147 | | 77 | | 45 | | 25 |  |  |
| Adjusted HR | 1.00 | | | 1.04 (0.70, 1.56) | | 0.91 (0.59, 1.41) | | 1.23 (0.75, 2.03) | | 1.33 (0.75, 2.34) | 1.04 (0.98, 1.10) | 0.11 |
|  |  | | |  | |  | |  | |  |  |  |
| Small intestinal cancer | | | | | | | | | | | | |
| Cases | 12 | | | 48 | | 22 | | 17 | | 9 |  |  |
| Adjusted HR | 1.00 | | | 1.34 (0.54, 3.31) | | 1.01 (0.38, 2.68) | | 2.16 (0.78, 5.97) | | 1.65 (0.48, 5.66) | 1.04 (0.91, 1.19) | 0.51 |
|  |  | | |  | |  | |  | |  |  |  |
| Colon cancer | | | | | | | | | | | | |
| Cases | 271 | | | 903 | | 518 | | 269 | | 116 |  |  |
| Adjusted HR | 1.00 | | | 0.86 (0.72, 1.02) | | 0.82 (0.68, 0.98) | | 0.94 (0.75, 1.16) | | 0.91 (0.70, 1.18) | 0.98 (0.94, 1.02) | 0.33 |
|  |  | | |  | |  | |  | |  |  |  |
| Rectal and anal cancer | | | | | | | | | | | | |
| Cases | 141 | | | 470 | | 273 | | 122 | | 75 |  |  |
| Adjusted HR | 1.00 | | | 0.86 (0.68, 1.08) | | 0.86 (0.68, 1.10) | | 0.81 (0.60, 1.09) | | 0.93 (0.66, 1.32) | 1.00 (0.96, 1.05) | 0.71 |
|  |  | | |  | |  | |  | |  |  |  |
| Liver cancer |  | | |  | |  | |  | |  |  |  |
| Cases | 41 | | | 110 | | 68 | | 27 | | 15 |  |  |
| Adjusted HR | 1.00 | | | 0.89 (0.56, 1.40) | | 0.90 (0.55, 1.48) | | 0.97 (0.54, 1.74) | | 0.72 (0.34, 1.50) | 0.95 (0.87, 1.05) | 0.31 |
|  |  | | | | | | | | | | | |
| Hepatocellular carcinoma | | | | | | | | | | | | |
| Cases | 23 | | | 54 | | 34 | | 12 | | 8 |  |  |
| Adjusted HR | 1.00 | | | 0.52 (0.29, 0.94) | | 0.56 (0.29, 1.07) | | 0.48 (0.21, 1.11) | | 0.44 (0.16, 1.20) | 0.88 (0.75, 1.02) | 0.09 |
|  |  | | |  | |  | |  | |  |  |  |
| Intrahepatic bile duct carcinoma | | | | | | | | | | | | |
| Cases | 13 | | | 42 | | 28 | | 11 | | <5 |  |  |
| Adjusted HR | 1.00 | | | 1.62 (0.65, 4.01) | | 1.65 (0.64, 4.26) | | 2.25 (0.83, 6.50) | | 0.44 (0.05, 3.72) | 0.99 (0.83, 1.15) | 0.86 |
|  |  | | |  | |  | |  | |  |  |  |
| Gall bladder and extrahepatic bile duct carcinoma | | | | | | | | | | | | |
| Cases | 13 | | | 66 | | 38 | | 17 | | 11 |  |  |
| Adjusted HR | 1.00 | | | 1.51 (0.72, 3.15) | | 1.44 (0.66, 3.11) | | 1.37 (0.55, 3.43) | | 2.18 (0.82, 5.75) | 1.05 (0.96, 1.17) | 0.25 |
|  |  | | |  | |  | |  | |  |  |  |
| Pancreatic cancer | | | |  | |  | |  | |  |  |  |
| Cases | 69 | | | 251 | | 143 | | 66 | | 42 |  |  |
| Adjusted HR | 1.00 | | | 0.91 (0.66, 1.25) | | 0.89 (0.63, 1.24) | | 0.87 (0.58, 1.30) | | 1.11 (0.70, 1.75) | 1.02 (0.96, 1.08) | 0.42 |
|  |  | | |  | |  | |  | |  |  |  |
| All digestive cancer | | | |  | |  | |  | |  |  |  |
| Cases | 650 | | | 2,206 | | 1,244 | | 620 | | 342 |  |  |
| Adjusted HR | 1.00 | | | 0.95 (0.85, 1.05) | | 0.90 (0.80, 1.01) | | 0.99 (0.87, 1.14) | | 1.09 (0.93, 1.27) | 1.01 (0.99, 1.03) | 0.24 |
|  | |  | |  | |  | | |  |  |  |  |

Model contains age at baseline, gender, deprivation, education, BMI, alcohol, smoking, fruit and vegetable intake, tea intake, physical activity, comorbidities at baseline (including high cholesterol, hypertension, diabetes, angina, myocardial infarction, stroke, peptic ulcer disease, hepatitis, cirrhosis, and gallstones).

| Supplementary Table 2: Sensitivity analysis by excluding two years after baseline for association between coffee type and digestive cancer within UK Biobank | | | | | |
| --- | --- | --- | --- | --- | --- |
|  |  | Most common type of coffee used | | | |
|  | Non-use | Decaffeinated | Instant | Ground | Other types |
| *n* | 103 560 | 68 756 | 200 045 | 82 196 | 6606 |
|  |  |  |  |  |  |
| Oesophageal cancer | | | | | |
| Cases | 58 | 42 | 135 | 39 | 6 |
| Adjusted HR | 1.00 | 1.38 (0.84, 2.27) | 1.27 (0.85, 1.90) | 1.21 (0.73, 1.99) | 1.58 (0.48, 5.16) |
|  |  |  |  |  |  |
| Oesophageal squamous cell carcinoma | | |  |  |  |
| Cases | 11 | 10 | 30 | 8 | <5 |
| Adjusted HR | 1.00 | 1.59 (0.50, 5.08) | 1.52 (0.59, 3.89) | 1.61 (0.52, 4.90) | 6.35 (1.27, 31.75) |
|  |  |  |  |  |  |
| Oesophageal adenocarcinoma | |  |  |  |  |
| Cases | 44 | 31 | 97 | 28 | 5 |
| Adjusted HR | 1.00 | 1.46 (0.82, 2.59) | 1.27 (0.79, 2.03) | 1.15 (0.63, 2.08) | 1.42 (0.33, 6.02) |
|  |  |  |  |  |  |
| Gastric cancer | | | | | |
| Cases | 52 | 20 | 97 | 25 | <5 |
| Adjusted HR | 1.00 | 0.87 (0.48, 1.56) | 1.12 (0.73, 1.72) | 0.92 (0.52, 1.61) | 1.16 (0.28, 4.88) |
|  |  |  |  |  |  |
| Small intestinal cancer | | | | | |
| Cases | 12 | 8 | 26 | 9 | <5 |
| Adjusted HR | 1.00 | 1.61 (0.52, 4.92) | 1.03 (0.38, 2.76) | 1.33 (0.43, 4.06) | 6.05 (1.21, 30.24) |
|  |  |  |  |  |  |
| Colon cancer | | | | | |
| Cases | 271 | 171 | 518 | 173 | 17 |
| Adjusted HR | 1.00 | 0.88 (0.70, 1.11) | 0.86 (0.71, 1.03) | 0.82 (0.65, 1.03) | 1.01 (0.55, 1.86) |
|  |  |  |  |  |  |
| Rectal and anal cancer | | | | | |
| Cases | 141 | 78 | 286 | 92 | 6 |
| Adjusted HR | 1.00 | 0.78 (0.56, 1.09) | 0.91 (0.71, 1.17) | 0.81 (0.60, 1.11) | 0.70 (0.26, 1.92) |
|  |  |  |  |  |  |
| Liver cancer | | | | | |
| Cases | 41 | 18 | 67 | 21 | <5 |
| Adjusted HR | 1.00 | 0.74 (0.37, 1.49) | 0.93 (0.57, 1.52) | 0.94 (0.50, 1.77) | 0.75 (0.10, 5.56) |
|  |  |  |  |  |  |
| Hepatocellular carcinoma | | | | | |
| Cases | 23 | 9 | 33 | 10 | <5 |
| Adjusted HR | 1.00 | 0.50 (0.19, 1.31) | 0.51 (0.27, 0.98) | 0.62 (0.26, 1.47) | Too small |
|  |  |  |  |  |  |
| Intrahepatic bile duct carcinoma | | | | | |
| Cases | 13 | 7 | 25 | 8 | <5 |
| Adjusted HR | 1.00 | 1.33 (0.40, 4.47) | 1.71 (0.66, 4.44) | 1.51 (0.46, 4.93) | 3.35 (0.40, 28.02) |
|  |  |  |  |  |  |
| Gall bladder and extrahepatic bile duct carcinoma | | | | | |
| Cases | 13 | 19 | 27 | 17 | <5 |
| Adjusted HR | 1.00 | 1.96 (0.82, 4.67) | 1.22 (0.54, 2.72) | 2.18 (0.88, 5.41) | Too small |
|  |  |  |  |  |  |
| Pancreatic cancer | | | | | |
| Cases | 69 | 36 | 150 | 58 | <5 |
| Adjusted HR | 1.00 | 0.59 (0.36, 0.96) | 0.97 (0.69, 1.35) | 1.11 (0.74, 1.67) | 0.64 (0.15, 2.62) |
|  |  |  |  |  |  |
| All digestive cancer |  |  |  |  |  |
| Cases | 650 | 392 | 1,295 | 429 | 41 |
| Adjusted HR | 1.00 | 0.90 (0.78, 1.05) | 0.96 (0.85, 1.08) | 0.94 (0.81, 1.08) | 0.99 (0.66, 1.49) |
|  |  |  |  |  |  |

Model contains age at baseline, gender, deprivation, education, BMI, alcohol, smoking, fruit and vegetable intake, tea intake, physical activities, comorbidities at baseline (including high cholesterol, hypertension, diabetes, angina, myocardial infarction, stroke, peptic ulcer disease, hepatitis, cirrhosis, and gallstones).

| Supplementary Table 3: The association between any coffee intake and risk of digestive cancer adjusting for additional smoking variable. | | |
| --- | --- | --- |
|  | Coffee intake | |
|  | No | Yes |
| *n* | 104 465 | 365 157 |
| Oesophageal cancer |  |  |
| Cases | 74 | 279 |
| Unadjusted HR | 1.00 | 0.97 (0.75, 1.25) |
| Adjusted HR | 1.00 | 1.27 (0.87, 1.86) |
|  |  |  |
| Oesophageal squamous cell carcinoma |  |  |
| Cases | 15 | 61 |
| Unadjusted HR | 1.00 | 1.05 (0.60, 1.86) |
| Adjusted HR | 1.00 | 1.12 (0.49, 2.54) |
|  |  |  |
| Oesophageal adenocarcinoma |  |  |
| Cases | 55 | 204 |
| Unadjusted HR | 1.00 | 0.94 (0.70, 1.27) |
| Adjusted HR | 1.00 | 1.49 (0.94, 2.36) |
|  |  |  |
| Gastric cancer |  |  |
| Cases | 65 | 184 |
| Unadjusted HR | 1.00 | 0.72 (0.55, 0.96) |
| Adjusted HR | 1.00 | 0.93 (0.64, 1.35) |
|  |  |  |
| Small intestinal cancer |  |  |
| Cases | 14 | 63 |
| Unadjusted HR | 1.00 | 1.18 (0.66, 2.11) |
| Adjusted HR | 1.00 | 1.85 (0.70, 4.88) |
|  |  |  |
| Colon cancer |  |  |
| Cases | 320 | 1141 |
| Unadjusted HR | 1.00 | 0.93 (0.82, 1.05) |
| Adjusted HR | 1.00 | 0.91 (0.77, 1.07) |
|  |  |  |
| Rectal and anal cancer |  |  |
| Cases | 176 | 610 |
| Unadjusted HR | 1.00 | 0.91 (0.77, 1.08) |
| Adjusted HR | 1.00 | 0.85 (0.68, 1.05) |
|  |  |  |
| Liver cancer |  |  |
| Cases | 48 | 134 |
| Unadjusted HR | 1.00 | 0.72 (0.52, 1.00) |
| Adjusted HR | 1.00 | 0.79 (0.50, 1.23) |
|  |  |  |
| Hepatocellular carcinoma |  |  |
| Cases | 26 | 62 |
| Unadjusted HR | 1.00 | 0.61 (0.39, 0.97) |
| Adjusted HR | 1.00 | 0.49 (0.27, 0.90) |
|  |  |  |
| Intrahepatic bile duct carcinoma |  |  |
| Cases | 16 | 56 |
| Unadjusted HR | 1.00 | 0.90 (0.52, 1.57) |
| Adjusted HR | 1.00 | 1.26 (0.56, 2.78) |
|  |  |  |
| Gall bladder and extrahepatic bile duct carcinoma | |  |
| Cases | 14 | 81 |
| Unadjusted HR | 1.00 | 1.48 (0.84, 2.61) |
| Adjusted HR | 1.00 | 11.49 (0.74, 3.00) |
|  |  |  |
| Pancreatic cancer |  |  |
| Cases | 81 | 303 |
| Unadjusted HR | 1.00 | 0.95 (0.75, 1.22) |
| Adjusted HR | 1.00 | 0.86 (0.63, 1.18) |
|  |  |  |
| All digestive cancer |  |  |
| Cases | 784 | 2767 |
| Unadjusted HR | 1.00 | 0.91 (0.84, 0.99) |
| Adjusted HR | 1.00 | 0.94 (0.63, 1.18) |
|  |  |  |

Model contains age at baseline, sex, deprivation, education, BMI, alcohol, smoking (never, ex stop>=20yrs, >=10-20yrs, ex stop<10yrs, current <=10 cigarettes per day, 10-20per day, >20per day), fruit and vegetable intake, tea intake, physical activity, comorbidities at baseline (including high cholesterol, hypertension, diabetes, angina, myocardial infarction, stroke, peptic ulcer disease, hepatitis, cirrhosis, and gallstones).

Supplementary Table 4: The association between coffee intake and hepatocellular carcinoma by sex, BMI, alcohol, and smoking status

|  | Unadjusted HR  (95% CI) | Adjusted HR  (95% CI) | P for interaction |
| --- | --- | --- | --- |
| Main analysis | 88 | 64 |  |
|  | 0.61 (0.39, 0.97) | 0.50 (0.29, 0.87) |  |
|  |  |  |  |
| Sex |  |  |  |
| Male | 71 | 53 |  |
|  | 0.54 (0.32, 0.89) | 0.48 (0.26, 0.86) | 0.21 |
| Female | 17 | 11 |  |
|  | 0.91 (0.29, 2.80) | 1.10 (0.21, 5.55) |  |
|  |  |  |  |
| BMI |  |  |  |
| Normal\under weight | 15 | 9 |  |
|  | 0.30 (0.10, 0.82) | 0.12 (0.02, 0.57) |  |
| Overweight | 36 | 28 |  |
|  | 0.49 (0.24, 0.99) | 0.46 (0.20, 1.04) | 0.056 |
| Obese | 37 | 27 |  |
|  | 1.2 (0.52, 2.73) | 0.86 (0.30, 2.41) |  |
|  |  |  |  |
| Alcohol consumption |  |  |  |
| Never | 17 | 12 |  |
|  | 1.20 (0.44, 3.27) | 0.24 (0.06, 0.95) |  |
| < 1 day per week | 20 | 13 |  |
|  | 0.96 (0.34, 2.65) | 0.73 (0.20, 2.62) |  |
| 1-2 days per week | 14 | 11 | 0.76 |
|  | 0.65 (0.20, 2.09) | 0.44 (0.11, 1.77) |  |
| 3-4 days per week | 17 | 12 |  |
|  | 0.82 (0.23, 2.87) | 0.88 (0.16, 4.59) |  |
| >4 days per week | 20 | 16 |  |
|  | 0.25 (0.10, 0.62) | 0.49 (0.15, 1.60) |  |
|  |  |  |  |
| Smoking status |  |  |  |
| Never | 30 | 19 |  |
|  | 0.56 (0.26, 1.19) | 0.66 (0.24, 1.81) |  |
| Previous | 40 | 31 |  |
|  | 0.54 (0.27, 1.08) | 0.44 (0.20, 0.99) | 0.83 |
| Current | 18 | 14 |  |
|  | 0.85 (0.28, 2.59) | 0.40 (0.11, 1.48) |  |
|  |  |  |  |

Model contains age at baseline, gender, deprivation, education, BMI, alcohol, smoking, fruit and vegetable intake, tea intake, physical activities, comorbidities at baseline (including high cholesterol, hypertension, diabetes, angina, myocardial infarction, stroke, peptic ulcer disease, hepatitis, cirrhosis, and gallstones).

Supplementary Figure 1: The dose response pattern of coffee consumption and digestive cancer

**Digestive cancer sites**

**Oesophageal cancer**

Any

>0 to 2

3 to 4

≥ 5

**Gastric cancer**

Any

>0 to 2

3 to 4

≥ 5

**Small intestinal cancer**

Any

>0 to 2

3 to 4

≥ 5

**Colon cancer**

Any

>0 to 2

3 to 4

≥ 5

**Rectal and anal cancer**

Any

>0 to 2

3 to 4

≥ 5

**Liver cancer**

Any

>0 to 2

3 to 4

≥ 5

**Hepatocellular carcinoma**

Any

>0 to 2

3 to 4

≥ 5

**Intrahepatic bile duct carcinoma**

Any

>0 to 2

3 to 4

≥ 5

**Adjusted HR, 95% CI**

1.15 [0.83, 1.59]

1.11 [0.77, 1.60]

1.08 [0.71, 1.64]

1.47 [0.94, 2.30]

0.98 [0.68, 1.41]

0.88 [0.59, 1.31]

1.14 [0.73, 1.78]

1.18 [0.70, 1.99]

1.39 [0.60, 3.22]

1.12 [0.49, 2.56]

1.67 [0.67, 4.16]

1.65 [0.56, 4.86]

0.91 [0.78, 1.06]

0.89 [0.76, 1.04]

0.97 [0.80, 1.18]

0.87 [0.68, 1.11]

0.88 [0.72, 1.08]

0.90 [0.72, 1.13]

0.81 [0.62, 1.06]

0.95 [0.70, 1.29]

0.87 [0.58, 1.31]

0.88 [0.57, 1.36]

0.87 [0.51, 1.48]

0.83 [0.45, 1.53]

0.50 [0.29, 0.86]

0.55 [0.30, 1.01]

0.44 [0.20, 0.97]

0.44 [0.17, 1.14]

1.49 [0.71, 3.13]

1.47 [0.68, 3.18]

1.80 [0.74, 4.38]

1.06 [0.32, 3.51]

**Adjusted HR, 95% CI**

0

Ref

0

Ref

0

0 3rrrrrrrrrrrrrrrrrrrrrrrrrrrrrrrrrrrrrrrrrrrrrrrrrrrrrrrrrrrrrrrrrrrrrrrrrrrrrrrrrrrrrrrrrrrrrrrrrrrrrrrrrrrrrrrrrrrrrrrrrrrrrrrrrrrrrrrrrrrrrrrrrrrrrrrrrrrrrrrrrrrrrrrrrrrrrrrrrrrrrrrrrrrrrrrrrrrrrrrrrrrrrrrrrrrrrrrrrrrrrrrrrrrrrrrrrrrrrrrrrrrrrrrrrrrrrrrrrrrrrrrrrrrrrrrrrrrrrrrrrrrrrrrrrrrrrrrrrrrrrrrrrrrrrrrrrrrrrrrrrrrrrrrrrrrrrrrrrrrrrrrrrrrrrrrrrrrrrrrrrrrrrrrrrrrrrrrrrrrrrrrrrrrrrrrrrrrrrrrrrrrrrrrrrrrrrrrrrrrrrrrrrrrrrrrrrrrrrrrrrrrrrrrrrrrrrrrrrrrrrrrrrrrrrrrrrrrrrrrrrrrrrrrrrrrrrrrrrrrrrrrrrrrrrrrrrrrrrrrrrrrrrrrrrrrrrrrrrrrrrrrrrrrrrrrrrrrrrrrrrrrrrrrrrrrrrrrrrrrrrrrrrrrrrrrrrrrrrrrrrrrrrrrrrrrrrrrrrrrrrrrrrrrrrrrrrrrrrrrrrrrrrrrrrrrrrrrrrrrrrrrrrrrrrrrrrrrrrrrrrrrrrrrrrrrrrrrrrrr20

0

0

0

0

Ref

Ref

Ref

Ref

Ref

**Gall bladder and extra**

3 to 4

Any

>0 to 2

0

≥ 5

1.63 [0.81, 3.28]

1.52 [0.74, 3.12]

1.56 [0.67, 3.63]

2.40 [0.98, 5.88]

**Pancreatic cancer**

3 to 4

≥ 5

Any

0

>0 to 2

3to 4

>0 to 2

≥ 5

0

Any

**All digestive cancer**

**hepatic bile duct carcinoma**

0.89 [0.66, 1.20]

0.86 [0.63, 1.17]

0.88 [0.60, 1.29]

1.08 [0.71, 1.64]

0.95 [0.86, 1.05]

0.92 [0.83, 1.02]

0.98 [0.87, 1.10]

1.05 [0.90, 1.22]

1

2

5

0.5

0.2

Ref

Ref

Ref

Ref

Supplementary Figure 2: Coffee consumption by type and risk of digestive cancer

**Digestive cancer sites**

**Decaffeinated vs. None**

Oesophageal cancer

Gastric cancer

Small intestinal cancer

Colon cancer

Rectal and anal cancer

Liver cancer

Hepatocellular carcinoma

Intrahepatic bile duct carcinoma

Gall bladder and extra hepatic bile duct carcinoma

Pancreatic cancer

**Instant vs. None**

Oesophageal cancer

Gastric cancer

Small intestinal cancer

Colon cancer

Rectal and anal cancer

Liver cancer

Hepatocellular carcinoma

Intrahepatic bile duct carcinoma

Gall bladder and extra hepatic bile duct carcinoma

Pancreatic cancer

**Ground vs. None**

Oesophageal cancer

Gastric cancer

Small intestinal cancer

Colon cancer

Rectal and anal cancer

Liver cancer

Hepatocellular carcinoma

Intrahepatic bile duct carcinoma

Gall bladder and extra hepatic bile duct carcinoma

Pancreatic cancer

**Adjusted HR**, **95% CI**

1.27 [0.82, 1.97]

0.88 [0.52, 1.49]

1.70 [0.65, 4.45]

0.95 [0.78, 1.16]

0.83 [0.62, 1.11]

0.82 [0.45, 1.49]

0.59 [0.25, 1.39]

0.97 [0.38, 2.48]

2.44 [1.10, 5.41]

0.66 [0.43, 1.01]

1.12 [0.78, 1.61]

1.04 [0.70, 1.55]

1.07 [0.46, 2.49]

0.91 [0.77, 1.08]

0.95 [0.76, 1.19]

0.91 [0.59, 1.40]

0.51 [0.28, 0.93]

1.27 [0.63, 2.56]

1.33 [0.63, 2.81]

0.95 [0.69, 1.31]

1.08 [0.78, 1.50]

0.87 [0.52, 1.46]

1.16 [0.42, 3.20]

0.84 [0.68, 1.04]

0.80 [0.60, 1.07]

0.84 [0.47, 1.50]

0.47 [0.20, 1.10]

1.46 [0.63, 3.38]

1.84 [0.77, 4.40]

0.99 [0.68, 1.44]

0.2

0.5

1

2

5

Increased risk

Decreased risk

**Adjusted HR**, **95% CI**
